# Genetic Variability in Key Genes in Prostaglandin E_2_ Pathway (*COX-2, HPGD, ABCC4* and *SLCO2A1*) and Their Involvement in Colorectal Cancer Development

**DOI:** 10.1371/journal.pone.0092000

**Published:** 2014-04-02

**Authors:** Carina Pereira, Sara Queirós, Ana Galaghar, Hugo Sousa, Pedro Pimentel-Nunes, Catarina Brandão, Luís Moreira-Dias, Rui Medeiros, Mário Dinis-Ribeiro

**Affiliations:** 1 Molecular Oncology Group, Investigation Centre, Portuguese Institute of Oncology, Porto, Portugal; 2 Abel Salazar Institute of Biomedical Sciences, University of Porto, Porto, Portugal; 3 Research Department, Portuguese League Against Cancer, Porto, Portugal; 4 Pathology Department, Portuguese Institute of Oncology, Porto, Portugal; 5 Gastroenterology Department, Portuguese Institute of Oncology, Porto, Portugal; 6 Physiology Department, Faculty of Medicine, University of Porto, Porto, Portugal; 7 CEBIMED, Faculty of Health Sciences of Fernando Pessoa University of Porto, Porto, Portugal; 8 CINTESIS/Department of Biostatistics and Medical Informatics, Faculty of Medicine, University of Porto, Porto, Portugal; University of Oslo, Norway

## Abstract

The pro-carcinogenic effects of prostaglandin E2 (PGE_2_) in colonic mucosa are not only regulated by the rates between Cyclooxygenase-2 (COX-2) biosynthesis and 15-Hydroxyprostaglandin Dehydrogenase (15-PGDH)-dependent degradation but also the steady-state levels of PGE_2_ in extracellular microenvironment, maintained by key specific prostaglandin transporters, the Multidrug Resistance Protein (MRP4) (efflux carrier) and Prostaglandin Transporter (PGT) (influx carrier). To understand the contribution of genetic variability in genes coding for COX-2/15-PGDH/MRP4/PGT proteins in CRC development, we conducted a hospital-based case-control study involving 246 CRC patients and 480 cancer-free controls. A total of 51 tagSNPs were characterized using the Sequenom platform through multiplexed amplification followed by mass-spectrometric product separation or allelic discrimination using real-time PCR. Seven tagSNPs were implicated in CRC development: the rs689466 in *COX-2* gene, the rs1346271 and rs1426945 in 15-PGDH, the rs6439448 and rs7616492 in PGT and rs1751051 and rs1751031 in MRP4 coding genes. Upon a stratified analysis a measurable gene-environment interaction was noticed between rs689466 and smoking habits, with individuals ever-smokers carriers of rs689466 GG homozygous genotype having a nearly 6-fold increased susceptibility for CRC onset (95%CI: 1.49–22.42, *P* = 0.011). Furthermore, the multifactor dimensionality reduction (MDR) analysis identified an overall four-factor best gene-gene interactive model, including the rs1426945, rs6439448, rs1751051 and rs1751031 polymorphisms. This model had the highest cross-validation consistency (10/10, *P*<0.0001) and an accuracy of 0.6957 and was further associated with a 5-fold increased risk for CRC development (95%CI: 3.89–7.02, *P*<0.0001). In conclusion, specific low penetrance genes in the pro-carcinogenic PGE_2_ pathway appear to modulate the genetic susceptibility for CRC development. A clearer understanding on CRC etiology through the identification of biomarkers of colorectal carcinogenesis might allow a better definition of risk models that are more likely to benefit from targeted preventive strategies to reduce CRC burden.

## Introduction

Colorectal cancer (CRC) is the most widespread malignancy in developed regions, accounting for over 13% of all diagnosed cases (728.550 cases) and 11% of all cancer-related deaths in 2008 (320.279 deaths) [1]. The burden of CRC is increasing as a reflection of population growth and aging, also as, an increased adoption of cancer-associated “westernized” lifestyle [2]. So, the implementation of population-based CRC screening guidelines focusing on the detection and removal of precancerous lesions is highly recommended for a successful decrease in CRC incidence rates [3]. Unfortunately, the compliance rates are far from the desirable and considerably lower than those reported for other recommended preventive strategies [4], which compromises the efficacy of these approaches in CRC prevention. This might provide reasoning not only for targeted screening but also the pursuit for alternative and/or complementary strategies, namely the use of chemoprevention to significantly reduce this cancer burden.

One group of compounds with extensive data supporting their preventive role in cancer onset include the nonsteroidal anti-inflammatory drugs (NSAIDs), shown to reduce the relative risk of developing CRC by 40–50%, mainly by targeting the cyclooxygenase-2 (COX-2) enzyme [5]–[7].

COX-2 is an immediate-early response gene, previously shown to be up-regulated in 40–50% of colorectal adenomas and 85% of CRC, leading to the extracellular microenvironment accumulation of prostaglandins (PGs) [8]. COX-2-derived PGE_2,_ the major PG produced in colorectal tumors, plays a key contribution to the hallmarks of cancer, by stimulating cell proliferation, invasiveness and migration, enhancing angiogenesis, evading apoptosis and modulating the antitumor immune response [9]. COX-2 has a physiologic antagonist in 15-hydroxyprostaglandin dehydrogenase (15-PGDH) that catabolizes PGE_2_ to an inactive keto compound [10]. 15-PGDH is highly expressed in normal mucosa and one of the most down-regulated genes in colorectal tumors, being a potent *in vivo* suppressor of colon neoplasia by decreasing the catabolism of PGE_2_ [11], [12]. Furthermore, low 15-PGDH levels are associated with resistance to COX-2 selective inhibitor Celecoxib chemopreventive effects in colorectal tumors development, reinforcing the impact of loss of 15-PGDH expression in colorectal carcinogenesis [13]. Notwithstanding, the biologic effects of the COX-2/PGE_2_ pathway are not only regulated by the rates between COX-2 biosynthesis and 15-PGDH-dependent degradation but also the steady-state levels of PGE_2_ in extracellular microenvironment, regulated by key specific prostaglandin transporters [14], [15]. The multidrug resistance-associated protein 4 (MRP4) is responsible for exporting PGE_2_ into the extracellular milieu, where a plethora of pathways will be activated through binding to specific G-protein couple receptors [14]. On the other hand, the active uptake back into the cytoplasm, where PGE_2_ will be inactivated by 15-HPGD, is carried-out by prostaglandin transporter (PGT) [15]. In fact, Holla and colleagues [16] reported that PGT and MRP4 mRNA levels are inversely regulated in human CRC, with PGT expression being downregulated and MRP4 overexpressed in CRC tissues and cell lines leading to higher levels of PGE_2_ extracellularly thus upregulating the effects of COX-2/PGE_2_ pathway.

A decade ago the release of the first human genome draft allowed a deeper knowledge on the architecture and function of the human genome, highlighting the relevance of common genetic variations on disease genesis. In CRC, family history is a well-established etiologic factor, shedding some clues for the involvement of low penetrance genes in its oncogenesis [17].

The *COX-2* gene is genetically polymorphic and was the target of several genetic association studies, implicating the involvement of three polymorphism in *COX-2* gene on colorectal tumors development (rs20417, rs699466 and rs5275, also known as −765G>C, −1195A>G and 8473T>C, respectively), although not always consistently [18]. In a preliminary study, we reported an increased susceptibility for CRC development in G allele carriers of the rs689466A>G polymorphism in *COX-2* promoter’s [19].

Hoeft and colleagues [20] firstly identified two tagging single nucleotide polymorphisms (tagSNPs), the rs8752 and rs2612656 in *HPGD* gene, coding for the 15-PGDH protein, as increased susceptibility markers for CRC development. More recently, Thompson and colleagues [21] observed a 40% increased risk associated with the rs2555639 SNP located at 17.74 kb upstream of the 5′UTR of *HPGD* gene that was further validated in the replication set.

With the exception of a two-phase case-control study in a Spanish population [22] no previous study inquired the role of common genetic variants in MRP4 and PGT coding genes (*ATP-Binding Cassette Sub-Family C Member 4* (*ABCC4*) and *solute carrier organic anion transporter family, member 2A1* (*SLCO2A1*), respectively) in CRC genesis. Neither addressed the combined effect of SNPs in these four genes with pivotal roles in modulating the levels of PGE_2_ extracellularly. So, in this case-control study we explored the associations of 51 common genetic variations in *COX-2/HPGD/ABCC4/SLCO2A1* PGE_2_ pathway genes with CRC onset.

## Materials and Methods

### Sample Size Estimation

We estimated that the sample size required to detect an Odds Ratio (OR) equal or superior to 1.70 is 200 patients and 400 controls (2∶1 ratio) to achieve a statistical power of 80%, with a significance level of 5%, for polymorphisms with a frequency superior to 15%. (Epi Info version 6, Centers for Disease Control, Atlanta, Georgia). Considering that r^2^, used to select the tagSNPs, is inversely related to the magnitude by which the sample size must be increased in a study design, for a r^2^ of 0.8 we needed to increase our sample size by 25%.

### Study Population

This non-matched hospital-based case-control study included 726 participants: 246 histologically confirmed CRC patients and 480 cancer-free controls, from the northern region of Portugal and recruited at the *Instituto Português de Oncologia do Porto* (IPO-Porto).

Written informed consent was obtained from all recruited participants before their inclusion in the study, according to the Declaration of Helsinki. This research project was approved by the Ethics Committee of the IPO-Porto (ref. 0084/08) and *Comissão Nacional de Protecção de Dados* (ref. 6619/2011) that is the Portuguese Data Protection Authority.

#### Control group

In this group, individuals between 50 and 75 years of age, without any clinical evidence of CRC or other oncologic malignancy were randomly recruited from the blood donor’s service at IPO-Porto between July 2005 and February 2008.

#### CRC patients group

Patients with histologically confirmed CRC newly diagnosed between January 2002 and September 2007 were enrolled in this study. These patients were selected from a colonoscopy database from the Gastroenterology Department, aged 50 to 75 years, without previous history of inflammatory bowel disease or hereditary syndromes and who were scheduled for a follow-up consult at *Serviço de Gastrenterologia* or *Unidade de Digestivos* at IPO Porto between March and May 2008.

Two hundred and forty seven CRC patients were included out of the 387 expected to be recruited. During the recruitment or afterwards by telephone interview patients were asked to recall their lifestyle habits (smoking behavior, BMI, etc) in the previous year of CRC diagnosis. Medical records were reviewed to extract the clinicopathological variables (stage, tumor grade, presence of synchronous and metachronous lesions) and to exclude misclassification bias.

### Sample Collection and Biological Processing

Blood samples were collected using standard venipuncture technique with EDTA containing tubes. DNA was extracted from peripheral blood leukocytes using the QIAamp DNA Blood Mini Kit (Qiagen, Hilden, Germany), following the manufacturer’s instructions.

For patients unable to provide a blood sample, the DNA was extracted from formalin fixed paraffin embedded (FFPE) blocks from the Pathology Department at our institute. Two to four 10 μm thickness section were used in each extraction depending on the size of tissue area (1.5–3 cm^2^). Briefly, the CRC tissue specimens from each glass slide were scraped, using a clean razor blade, into a 1,5-ml microcentrifuge tube. The samples were deparaffinised in xylene for 10 minutes, at room temperature, followed by centrifugation at 14.000 g–16.000 g for 3 minutes. The tissue pellets were then rehydrated with 1 ml of absolute ethanol, followed by centrifugation at 14.000 g–16.000 g for 3 minutes and the supernatant was discarded. This step was repeated twice. Then, the tube was maintained open for 15 minutes to evaporate any remaining ethanol. Further steps of DNA isolation were performed using the GRS Genomic DNA Kit – Tissue, in accordance with the manufacturer’s protocol (GRiSP, Porto, Portugal).

DNA was quantified using the NanoDrop 1000 Spectrophotometer (Thermo Fisher Scientific, Wilmington, DE, USA) and stored at −20°C until genotype examination. The DNA quality was determined by measuring the optical density (OD) 260/280 ratio.

### Validation of DNA Genotyping Extracted from FFPE Samples

To assess whether DNA isolated from FFPE sections is reliable for retrospective genotyping we compared the genotypes of 20 somatic DNAs extracted from FFPE specimens to germline DNAs isolated from fresh peripheral blood from the same patients. The genotypes were highly concordant (100%).

### Polymorphisms Selection

Using a tagSNP approach, the genetic variants were retrieved from a set of common SNPs in the Caucasian population of HapMap project (CEU). The Genome Variation Server (version 7.00) was used to recover tagSNPs capturing variations (1) with a minor allele frequency equal or superior to 15%; (2) within the coding region of the genes plus 2 Kb upstream and downstream and (3) with a r^2^ superior to 0.8. A total of 140 tagSNPs were captured: 6, 15, 31 and 88 tagSNPs in *COX-2*, *HPGD*, *SLCO2A1* and *ABCC4* genes, respectively. We further selected SNPs with high likelihood of genotyping success using the Sequenom platform, (Sequenom, San Diego, CA). Briefly, tagSNPs were prioritized as follows: first, all non-singletons tagSNPs or singletons with expected functional repercussion (FuncPred software) were tested. TagSNPs with low genotyping scores were replaced with representative variants; and finally the non-significant singletons were included in the array design. A total of 55 SNPs were successfully converted to the Sequenom platform.

Furthermore, we also included polymorphisms that were previously associated with colorectal tumors development and had a minor allele frequency equal or superior to 15% that failed to converted to the Sequenom platform: rs20417, rs689466 and rs5275 in *COX-2* and rs2612656 and rs2555639 in *HPGD* genes.

### Genotype Characterization

TagSNP genotyping was performed using MassARRAY iPLEX Gold technology (Sequenom, San Diego, CA) based on multiplexed amplification followed by mass-spectrometric product separation. This technique was carried-out by the *Unidade de Genómica/Serviço de Genotipagem do Instituto Gulbenkian de Ciência.*

All polymorphisms not included in the tagSNPs analysis were characterized through allelic discrimination (Real-Time Polymerase Chain Reaction) using validated TaqMan® SNP genotyping assays (C___2517145_20, C___7550203_10, C___15909858_20, C___16038735_10 for the rs689466, rs5275, rs2612656 and rs2555639, respectively) with the exception of the polymorphism −765G>C (rs20417) which was custom designed (Applied Biosystems, Foster City, California USA). Allelic discrimination was performed by measuring end-point fluorescence using ABI PRISM Sequence Detection System (Applied Biosystems, Foster City, California USA).

### Quality Control

Genotypes were excluded from the analysis if any of the following criteria was applied: call rate inferior to 0.90; concordance rate inferior to 0.95 and Hardy-Weinberg equilibrium (HWE) with *P*<0.05. Blank templates were included in each 96 and 384-well plates to ensure contamination-free results. Two researchers performed the genotype interpretation independently and five to ten percent of all samples were randomly selected and re-submitted to a new genetic characterization to confirm the genotypes.

### Statistical Analysis

For genetic distribution analysis, the Hardy–Weinberg equilibrium was tested by the Pearson’s goodness-of-fit test to compare the observed versus the expected genotype distribution among the control population.

Data analysis was performed using the computer software IBM Statistical Package for Social Sciences-SPSS (IBM Corp., Armonk, New York, USA) for Macintosh (version 19.0). Chi-square analysis was used to compare categorical variables, using a 5% level of significance. Non-parametric tests were used to compare mean values. Odds ratio (OR) and its 95% confidence interval (CI) were calculated as a measure of the association between the genetic variants and the risk for the development of CRC. Covariates proven to differ between group populations were included in the logistic regression analysis. Gene-environment interaction analysis were carried-out by stratifying data considering the gender, smoking habits and body mass index (BMI). Additionally, a bootstrap resampling was used to investigate the stability of risk estimates (1000 replications). Furthermore, the false positive report probability (FPRP) was used to confirm the noteworthiness of significant findings, according to the study by Wacholder and colleagues [23]. The FPRP threshold was set at 0.5 under an assigned prior probability ranging from 0.01 to 0.25 to detect an OR of 1.5.

Haplotype analysis was performed at a gene level using the SNPStats software (www. http://bioinfo.iconcologia.net/SNPstats). The haplotype frequencies were estimated using the implementation of the EM algorithm coded into the *haplo.stats* package. The most frequent haplotype was automatically selected as the reference category. For the *HPGD*, *SLCO2A1* and *ABCC4* genes the haplotype blocks were constructed considering the most meaningful polymorphisms.

The open-source multifactor dimensionality reduction (MDR) software (version 3.0.2) (www.epistasis.org) was used to assess potential gene-gene interactions between SNPs with statistical significant impact on CRC genetic susceptibility. The fitness of an MDR model was estimated by determining the testing accuracy and its cross-validation consistency (CVC). Using a 10-fold cross-validation method the data was divided into 10 sets, in which 9 subsets were training sets and one subset was a test set. Hence, the CVC is a measure of the number of times of 10 divisions of the dataset the best model was extracted. The single best model normally has the maximal testing accuracy and CVC. Statistical significance was evaluated using a 1000-fold permutation test to compare observed testing accuracies with those expected under the null hypothesis of null association. Permutation testing corrected for multiple testing by repeating the entire analysis on 1000 datasets that were consistent with the null hypothesis.

## Results

### Description of Study Population

The characteristics of the study population are summarized in Table 1. Cases were significantly older than controls with a median age of 63 years (50–75) (vs. 58 years in controls (50–69), *P*<0.001). Males were overrepresented in both groups (60.1% vs 65.4% in cases and controls, respectively, *P* = 0.159) and nearly 77% of participants were overweight (*P* = 0.955). The majority of participants had also never smoked in either category (37.4% in cases and 39.7% in controls, *P* = 0.636).

**Table 1:** Description of participants.

	Cases	Controls	
	(n = 246)	(n = 480)	*P* value
Demographics			
*Age* (years)			
Mean (SD)	63 (7.2)	58 (4.9)	<0.001
Median (min–max)	63 (50–75)	58 (50–69)	
*Sex*, n (%)			
Male	146 (60.1)	314 (65.4)	0.159
Female	97 (39.9)	166 (34.6)	
Lifestyle behavior			
*BMI* (Kg/m^2^)			
Mean (SD)	28 (4.2)	28 (3.6)	0.510
Median (min–max)	28 (20–43)	27 (20–41)	
BMI category, n (%)#			
<25	34 (23.4)	48 (23.2)	0.955
≥25	111 (76.6)	159 (76.8)	
*Smoking status*, n (%)			
Never-smokers	92 (62.6)	219 (60.3)	0.636
Ever-smokers*	55 (37.4)	144 (39.7)	
Tumor characteristics			
*Tumor location*, n (%)			
Rectum	127 (52.3)	–	
Colon	116 (47.7)	–	
*Stage*, n (%)			
I–II	121 (52.6)	–	
III–IV	109 (47.4)	–	
*Grade*, n (%)			
Low grade	135 (95.7)	–	
High grade	6 (2.4)	–	
*Synchronous tumors*, n (%)			
Yes	14 (5.5)	–	
No	224 (88.2)	–	

BMI, body mass index;

# Categorization based on the cutoff defined by the world Health Organization for overweight people;

*Never- and former-smokers pooled together; For synchronous tumors the most advanced lesions was the one considered in the tumors’ characterization.

### Genotype Frequencies and Risk Estimates

Three SNPs and four samples were excluded from the analysis due to genotyping failure and four SNPs were dropped because their frequencies deviated from HWE (*P*<0.05). A total of 51 SNPs were included in the risk estimate analysis. The mean genotype call and concordance rates were 99.02% and 99.3%, respectively. The description of selected SNPs is displayed in Table S1.

Overall seven genetic polymorphisms across the four genes were implicated in colorectal carcinogenesis, as can be observed in Table 2. The AG and GG genotypes of the rs689466 polymorphism in *COX-2* gene were overrepresented in the group of cases leading to an increased risk for CRC more noticeable for homozygous GG although this was not statistically significant in the multivariate analysis (OR = 2.01; 95%CI:0.93–4.35, *P* = 0.076). The rs1346271 and rs1426945 SNPs in *HPGD* gene were associated with a 32% and 44% decreased risk for CRC onset (95%CI:0.47–0.96, *P* = 0.029 and 95%CI:0.34–0.93, *P* = 0.026, for the GC and AA homozygous carriers of the rs1346271 and rs1426945 polymorphisms, respectively). Out of the fifteen genetic variations analyzed in the *SLCO2A1* gene only the rs6439448 and rs7616492 polymorphisms influenced the susceptibility for CRC. Individuals carriers of the rs6439448 heterozygous AG genotype presented an OR of 0.68 (95%CI:0.47–0.99, *P* = 0.047). On the other hand, a two-fold increased predisposition was noticed for individuals carrying both copies of the A allele of rs7616492 polymorphism (95%CI:1.27–3.32, *P* = 0.003). Focusing on *ABCC4* gene, a 1.76 enhanced susceptibility was observed with the AA genotype of rs1751051 SNP and a protection was evident for AG genotype carriers of rs1751031 polymorphism (OR = 0.68; 95%CI:0.47–0.97, *P* = 0.032). The bootstrap analysis supported our results (Table 2). The genotypes distribution of all included SNPs is reported in Table S2.

**Table 2:** Genotype frequencies among cases and controls and risk estimates for the involvement of *COX-2/HPGD/SLCO2A1/ABCC4* polymorphisms in colorectal cancer onset.

SNP rs	Cases n (%)	Controls n (%)	Univariate analysis			Multivariate analysis			
			OR	95%CI	P value	aOR	95%CI	P value	P bootstrap
*COX-2*									
rs689466									
AA	143 (58.8)	323 (68.4)	1.00	Reference	–	1.00	Reference	–	–
AG	85 (35.0)	133 (28.2)	**1.44**	**1.03–2.02**	**0.032**	**1.53**	**1.08–2.17**	**0.018**	**0.028**
GG	15 (6.2)	16 (3.4)	**2.12**	**1.02–4.40**	**0.040**	2.01	0.93–4.35	0.076	0.063
*HPGD*									
rs1346271									
GG	104 (42.4)	174 (36.2)	1.00	Reference	–	1.00	Reference	–	–
GC	97 (39.6)	246 (51.2)	**0.66**	**0.47–0.92**	**0.016**	**0.68**	**0.47–0.96**	**0.029**	**0.034**
CC	44 (18.0)	60 (12.5)	1.23	0.78–1.94	0.382	1.34	0.83–2.17	0.231	0.260
rs1426945									
GG	110 (44.7)	169 (35.3)	1.00	Reference	–	1.00	Reference	–	–
GA	108 (43.9)	233 (48.6)	**0.71**	**0.51–0.99**	**0.044**	0.70	0.50–1.00	0.050	0.055
AA	28 (11.4)	77 (16.1)	**0.56**	**0.34–0.92**	**0.021**	**0.56**	**0.34–0.93**	**0.026**	**0.035**
*SLCO2A1*									
rs6439448									
CC	174 (72.2)	319 (66.6)	1.00	Reference	–	1.00	Reference	–	–
CA	56 (23.2)	143 (29.9)	0.72	0.50–1.03	0.071	**0.68**	**0.47–0.99**	**0.047**	**0.039**
AA	11 (4.6)	17 (3.5)	1.19	0.54–2.59	0.668	0.93	0.39–2.20	0.869	0.851
rs7616492									
GG	89 (37.1)	202 (42.2)	1.00	Reference	–	1.00	Reference	–	–
GA	103 (42.9)	216 (45.1)	1.08	0.77–1.52	0.651	1.18	0.82–1.69	0.373	0.369
AA	48 (20.0)	61 (12.7)	**1.79**	**1.14–2.81**	**0.012**	2.05	**1.27–3.32**	**0.003**	**0.002**
*ABCC4*									
rs1751051									
TT	111 (46.2)	234 (48.8)	1.00	Reference	–	1.00	Reference	–	–
TA	91 (37.9)	202 (42.1)	0.95	0.68–1.33	0.763	1.06	0.74–1.50	0.764	0.758
AA	38 (15.8)	44 (9.2)	**1.82**	**1.12–2.97**	**0.016**	**1.76**	**1.04–2.95**	**0.034**	0.053
rs1751031									
AA	164 (66.9)	298 (62.2)	1.00	Reference	–	1.00	Reference	–	–
AG	66 (26.9)	166 (34.7)	0.72	0.51–1.02	0.063	**0.68**	**0.47–0.97**	**0.032**	**0.031**
GG	15 (6.1)	15 (3.1)	1.82	0.87–3.81	0.111	1.67	0.77–3.63	0.194	0.168

a Odds ratio (OR) adjusted for age (categorical variable, using the global median age of 60 years as cutoff); CI, confidence interval; bootstrap results are based in 1000 samples.

Statistical significant results are at bold.

The FPRP analysis revealed that the unadjusted significant associations observed in Table 2, retained their significance (FPRP≤0.5) when a prior probability equal or superior to 0.10 was considered, with the exception of the rs689466 polymorphism (GG vs AA) that presented an FPRP of 0.690, suggesting possible bias in this positive finding (data not shown).

### Gene-environment Interaction Analysis

Upon a stratified analysis we observed, that with the exception of rs6439448 and rs1751051 polymorphisms in *SLCO2A1* and *ABCC4* gene, respectively all other variants appear to have a sex-dependent behavior particularly relevant in male carriers of GG genotype of *COX-2* rs689466 SNP (OR = 3.3; 95%CI:1.23–9.09, *P* = 0.018) and AA homozygous for the rs1426945 polymorphism in *HPGD* gene (OR = 0.38; 95%CI:0.20–0.74, *P* = 0.004), as reported in Table 3.

**Table 3:** Risk estimates for the involvement of polymorphisms in *COX-2/HPGD/SLCO2A1/ABCC4* genes in colorectal cancer onset stratified by sex, smoking habits and body mass index.

Gene	n	aOR	95%CI	*P* value	*P_bootstrap_*
*COX-2*					
rs689466 (AAvsGG)					
*Sex*					
Female	180	0.89	0.25–3.23	0.862	0.840
Male	313	3.34	1.23–9.09	**0.018**	**0.004**
*Smoking habits*					
Never-smokers	213	0.63	0.13–3.08	0.564	0.429
Ever-smokers*	142	5.77	1.49–22.42	**0.011**	**0.004**
*BMI* (kg/m^2^)^#^					
<25	59	3.63	0.20–64.59	0.381	0.071
≥25	182	2.41	0.72–8.07	0.154	0.123
*HPGD*					
rs1346271 (GGvsGC)					
*Sex*					
Female	214	0.42	0.23–0.78	**0.005**	**0.007**
Male	401	0.86	0.55–1.33	0.487	0.500
*Smoking habits*					
Never-smokers	271	0.88	0.51–1.51	0.644	0.613
Ever-smokers*	171	0.62	0.30–1.27	0.190	0.183
*BMI* (kg/m^2^)^#^					
<25	69	0.57	0.19–1.69	0.312	0.312
≥25	234	0.69	0.40–1.19	0.185	0.186
rs1426945 (GGvsAA)					
*Sex*					
Female	140	1.20	0.52–2.79	0.672	0.677
Male	211	0.38	0.20–0.74	**0.004**	**0.006**
*Smoking habits*					
Never-smokers	168	1.02	0.50–2.08	0.966	0.969
Ever-smokers*	101	0.83	0.31–2.21	0.705	0.730
*BMI* (kg/m^2^)^#^					
<25	42	1.07	0.28–4.12	0.922	0.932
≥25	135	1.29	0.59–2.86	0.525	0.563
*SLCO2A1*					
rs6439448 (CCvsCA)					
*Sex*					
Female	253	0.72	0.39–1.33	0.292	0.314
Male	433	0.66	0.41–1.07	0.094	0.082
*Smoking habits*					
Never-smokers	302	0.72	0.41–1.28	0.269	0.265
Ever-smokers*	185	0.64	0.29–1.41	0.272	0.277
*BMI* (kg/m^2^)^#^					
<25	79	1.04	0.34–3.13	0.95	0.947
≥25	258	0.75	0.42–1.34	0.34	0.312
rs7616492 (GGvsAA)					
*Sex*					
Female	135	1.60	0.72–3.58	0.250	0.254
Male	260	2.35	1.28–4.28	**0.005**	**0.008**
*Smoking habits*					
Never-smokers	163	1.48	0.64–3.27	0.37	0.382
Ever-smokers*	119	1.27	0.51–3.19	0.60	0.628
*BMI* (kg/m^2^)^#^					
<25	50	0.06	0.006–0.69	**0.023**	**0.012**
≥25	142	2.18	1.00–4.77	0.051	0.050
*ABCC4*					
rs1751051 (TTvsAA)					
*Sex*					
Female	151	2.20	0.83–5.81	0.111	0.151
Male	269	1.70	0.91–3.16	0.096	0.100
*Smoking habits*					
Never-smokers	179	2.32	1.05–5.13	**0.037**	**0.033**
Ever-smokers*	114	1.26	0.44–3.57	0.665	0.681
*BMI* (kg/m^2^)^#^					
<25	46	3.57	0.39–32.52	0.260	0.114
≥25	157	1.82	0.86–3.89	0.120	0.145
rs1751031 (AAvsAG)					
*Sex*					
Female	249	0.51	0.28–0.94	**0.030**	**0.031**
Male	437	0.78	0.49–1.22	0.269	0.269
*Smoking habits*					
Never-smokers	301	0.69	0.39–1.21	0.196	0.203
Ever-smokers*	193	0.76	0.38–1.52	0.439	0.454
*BMI* (kg/m^2^)^#^					
<25	80	0.52	0.19–1.45	0.213	0.255
≥25	261	0.76	0.44–1.31	0.324	0.351

a Odds ratio (OR) adjusted for age (categorical variable, using the global median age of 60 years as cutoff); CI, confidence interval; BMI, body mass index; ^#^Categorization based on the cutoff defined by the world Health Organization for overweight people;

*Never- and former-smokers pooled together.

Statistical significant results are at bold.

Furthermore, a nearly 6-fold increased risk was observed in ever-smokers carrying the GG genotype for the *COX-2* rs689466 polymorphism (95%CI:1.49–22.42, *P* = 0.011 vs OR = 0.63; 95%CI:0.13–3.08, *P* = 0.56 in never-smokers). In contrast, the *ABCC4* rs1751051 AA genotype seemed to lead to a higher susceptibility in individuals who never smoked (OR = 2.32; 95%CI:1.05–5.13, *P* = 0.037). The rs7616492 homozygous AA genotype of *SLCO2A1* gene played opposing roles when considering the interaction with BMI (OR = 0.06; 95%CI:0.01–0.69, *P* = 0.023 and OR = 2.18; 95%CI:1.00–4.77, *P* = 0.051 for individuals with BMI <25 and overweight (BMI≥25 kg/m^2^), respectively).

### Haplotype Analysis

Four common haplotypes were described for *COX-2* gene, as can be observed in Table 4. The most frequent haplotype, the AGT, was present in 52% of controls and used as the reference one. The block containing the rs689466 G allele, GGT, was associated with a 51% increased susceptibility consistent with the individual SNP analysis (95%CI:1.10–2.06, *P* = 0.010). Although we did not noticed any influence of rs5275 C allele in CRC risk independently, carriers of AGC haplotype had a 1.53–fold higher predisposition for CRC (95%CI:1.13–2.19, *P* = 0.008). The AGAC haplotype of *HPGD* gene was the most common (30%) out of the five blocks. An enhanced risk was observed for Individuals carrying the blocks, AGGC and ACGC, containing the rs1426945 G (OR = 1.70; 95%CI:1.22–2.37, *P* = 0.002 and OR = 1.60; 95%CI:1.04–2.44, *P* = 0.031, respectively). Coherently, the opposing rs1426945 AA genotype conferred a 40% risk reduction in the SNP analysis. The haplotype TAGAAC of *SLCO2A1* gene containing the decreased risk associated rs6439448 A allele and rs7616492 G allele led to a nearly 50% protection for CRC development compared with individuals carrying the TCAAAC reference block. The only common haplotype encompassing the rs1751051 A allele of *ABCC4* gene (AATTA) increased the susceptibility for CRC onset by over two-folds in contrast with the TATTA most frequent haplotype. No block contained the rs1751031 G allele.

**Table 4:** Haplotype frequencies between patients and controls and risk estimates for their involvement in colorectal cancer development.

Gene/Haplotype	% Cases	% Controls	aOR	95%CI	*P*
*COX-2* €					
A-G-T	44.9	52.4	1	Reference	–
G-G-T	21.9	17.3	1.51	1.10–2.06	**0.010**
A-G-C	18.3	13.4	1.57	1.13–2.19	**0.008**
A-C-C	10.3	15.1	0.82	0.56–1.20	0.310
*HPGD* *					
A-G-A-C	23.8	30.5	1	Reference	–
A-G-G-C	25.4	17.8	1.70	1.22–2.37	**0.002**
A-C-G-T	17.9	20.1	1.12	0.77–1.62	0.550
A-C-G-C	12.8	10.8	1.60	1.04–2.44	**0.031**
G-G-A-C	6.4	8.0	1.05	0.57–1.92	0.880
*SLCO2A1* £					
T-C-A-A-A-C	25.7	26.1	1	Reference	–
T-A-G-A-A-C	8.3	12.7	0.54	0.33–0.82	**0.012**
C-C-G-A-A-C	9.6	10.6	0.86	0.54–1.36	0.520
T-C-G-A-A-C	6.8	9.0	0.75	0.44–1.25	0.270
T-C-G-A-G-C	6.5	8.7	0.68	0.40–1.15	0.150
*ABCC4* ¥					
T-A-T-T-A	10.9	12.0	1	Reference	–
T-A-T-C-A	7.9	12.0	1.07	0.49–2.34	0.860
T-G-T-T-A	7.8	10.1	1.13	0.57–2.24	0.740
A-A-T-T-A	11.6	7.5	2.28	1.12–4.67	**0.024**
T-G-C-T-A	8.5	4.0	1.58	0.67–3.68	0.290
T-G-C-C-A	4.5	6.4	0.88	0.40–1.96	0.760

a Odds ratio (OR) adjusted for age (categorical variable, using the global median age of 60 years as cutoff); CI, confidence interval.

€ SNPs order: rs689466-rs20417-rs5275.

*SNPs order: rs2612656-rs1346271-rs1426945-rs12500316.

£ SNPs order: rs4241362-rs6439448-rs7616492-rs7625035-rs1131598-rs10935090.

¥ SNPs order: rs1751051-rs2274403-rs1678405-rs1678396-rs1751031.

### Gene-gene Interaction Analysis

An exhaustive MDR analysis was carried-out to evaluate all possible combinations of rs689466, rs1346271, rs1426945, rs6439448, rs7616492, rs1751051 and rs1751031 polymorphisms proven to be associated with CRC onset in the individual SNP analysis. As shown in Table 5, we observed the highest CVC (10/10) and accuracy (0.6957) in the four-factor interaction model, which shows an interaction between rs1426945 *HPGD* polymorphism, rs6439448 *SLCO2A1* SNP and rs1751051 and rs1751031 polymorphisms in *ABCC4* gene. This gene-gene interaction was associated with a 5-fold increased risk for CRC development (95%CI:3.89–7.02, *P*<0.0001).

**Table 5:** MDR analysis for the colorectal cancer risk prediction.

Best model	CV accuracy	CV consistency	OR	95%CI	*P*
rs1346271, rs1426945	0.6113	10/10	2.53	1.91–3.35	<0.0001
rs1426945,rs6439448, rs1751031	0.6376	6/10	3.19	2.39–4.28	<0.0001
rs1426945,rs6439448, rs1751051, rs1751031	0.6957	10/10	5.23	3.89–7.02	<0.0001

MDR, multifactor dimensionality reduction; CV, cross-validation; OR, odds ratio; CI, confidence interval.

## Discussion

Early screening and follow-up of individuals previously diagnosed with colorectal adenomas is the cornerstone of CRC prevention [3]. Nevertheless, the compliance rates in countries with implemented population-based CRC screening guidelines are far from the desirable for a successful impact in CRC incidence [4]. Although the regular use of NSAIDs has been consistently effective in the primary prevention of colorectal tumors its use is currently compromised by the onset of serious gastrointestinal side effects in average-risk population [24]. So, the challenge falls in the identification of biomarkers that could target higher-risk populations for colorectal screening and/or chemopreventive strategies.

In this case-control study we assessed the involvement of 51 tagSNPs in four genes (*COX-2/HPGD/SLCO2A1/ABCC4*) with key roles in PGE_2_ pathway in CRC development. Our results indicate that seven genetic polymorphisms are implicated in colorectal carcinogenesis: the rs689466A>G in *COX-2*, the rs1346271G>C and rs1426945G>A in *HPGD*, the rs6439448C>A and rs7616492G>A in *SLCO2A1* and the rs1751051T>A and rs1751031A>G in *ABCC4* gene.

The rs689466A>G in *COX-2* gene had a synergetic effect in CRC oncogenesis that increased with allele dosage, further reinforcing its causative role in cancer development. The GG homozygous genotype enhanced the susceptibility for CRC onset by 2-fold and appeared to have a sex and smoking habits dependent behavior, with ever-smokers having a nearly 6-fold increased genetic predisposition for CRC. These data follow our previous observations from a preliminary study [19]. Furthermore, two haplotypes containing either the rs689466G (GGT) or the rs5275C alleles (AGC) led to a 50% increase on the risk for CRC. The lack of consistency observed between epidemiological studies addressing the rs689466A>G SNP in different ethnic backgrounds or cancer models appears to suggest that not only population stratification and lifestyle habits might modulate this polymorphism behavior but also its influence might be cell, tissue and pathological condition-dependent [19], [25]–[29]. In fact, in a recently published study we reported that this polymorphism located at −1195 nucleotides upstream exon 1 increases *COX-2* transcriptional activity in two colon cancer cell lines [30]. This was also noticeable in human hepatoma cell lines [31] but antagonizes the increased promoter activity observed for the rs689466 A allele in gastric cancer cell lines [25]. COX-2 overexpression is suggested as one of the smoke-induced pathways involved in carcinogenesis [32], [33]. Tobacco contains more than 60 identified carcinogens and even though some, such as, nicotine and benzo[a]pyrene, were shown to trigger COX-2 expression through *b*-adrenoceptors and ERK1/2 pathways, respectively, the pathogenesis of smoking related CRC is still understudied [34]. Further functional studies are needed to elucidate the nature of this gene-environment interaction.

The rs5275T>C polymorphism, set at 8473 base pairs from exon 1 was previously associated with an increased risk for colorectal adenoma and here with a higher susceptibility for CRC in the context of the AGC haplotype (vs AGT) [18]. This T-to-C substitution in the 3′UTR was proven to contribute to COX-2 overexpression by disrupting the miR-542-3p:mRNA interaction and thus decreasing COX-2 mRNA decay [35].

As already mentioned, COX-2 has a predominant role in the synthesis of the pro-carcinogenic PGE_2_ bioactive lipid and the main molecular target of NSAIDs. In fact Chan and colleagues [36] noticed that aspirin’s preventive role was exclusively effective in the subgroup of colon cancers overexpressing COX-2 enzyme. So, the genetic variability in *COX-2* gene may help predict individuals at higher risk and expected to be exposed to higher levels of COX-2.

The expression and activity of 15-PGDH is repressed in colorectal cancer and Apc^min^ mouse adenomas, leading to a decrease in PGE_2_ catabolism, local tissue accumulation of PGE_2_ and resistance to Celecoxib chemoprevention in colon tumors [11], [13].

We were not able to reproduce in our population the associations reported in previous studies [20], [21], [37]. This could be attributed to population stratification involving differences in genetic ancestry as the study developed by Hoeft and colleagues [20] involved participants from 10 different European countries or these variants could be in linkage disequilibrium with a causative SNP with a lower allele frequency (<15%) thus limiting our statistical power to detect a true association. Nevertheless, we observed for the first time that the rs1346271G>C and rs1426945G>A tagSNPs in *HPGD* gene were associated with a decrease risk for CRC development. Both of these genetic variations are located in the 5′UTR of *HPGD* gene altering the transcription factors binding sites as predicted by the SNPinfo software (www.snpinfo.niehs.nih.gov) that ultimately could lead to a differential expression of 15-PGDH. Remarkably, inherited mutations in *HPGD* gene are linked to the development of primary hypertrophic osteoarthropathy (PHO), thus reinforcing the impact that the genetic variability in *HPGD* might portray in disease development by disrupting the normal 15-HPGD levels or activity [38].

To the best of our knowledge this is the first study addressing the involvement of these specific genetic polymorphisms in *SLCO2A1* and *ABCC4* genes, coding for the PGT and MRP4 specific prostaglandin transporters, in disease development. The efflux-dominated flow of PGE_2_ in neoplastic tumors, due to an increased in COX-2 and MRP4 and repressed expression of 15-HPGD and PGT is associated with high levels of PGE_2_ in the extracellular milieu culminating in the activation of a plethora of pathways that potentiate tumor development [8], [11], [16]. The rs6839448C>A and rs7617492G>A tagSNPs in *SLCO2A1* were implicated in colorectal carcinogenesis Furthermore, individuals carrying the haplotype containing both the A and G alleles of rs6839448 and rs7617492 tagSNPs (TAGAAC), respectively, had a nearly 50% protection for CRC. Although, the rs6439448 is not expected to be functional it tags two SNPs with predicted impact on PGT expression: the rs2370512T>A located in the 3′UTR that could affect the binding of microRNAs and stability of mRNA and the nonsynonymous rs34550074G>A SNP at codon 396 that codes for two different amino acids (Alanine>Threonine) with potential repercussion on protein structure and function.

Focusing on *ABCC4* gene, two tagSNPs influenced the genetic susceptibility for the development of CRC (rs1751051 and rs1751031), although none of the SNPs in the LD blocks tagged by these two SNPs could explain the altered risk for cancer development.

Common diseases have proven to be much more challenging to understand, as they are thought to arise due to the synergetic effect of many different susceptibility DNA variants interacting with environmental factors. Although, we have noticed some interactions between the aforementioned tagSNPs and demographic/lifestyle habits, the lack of complete characterization of the study population, decreased the statistical power and the scarcity of studies inquiring the influence of those environmental factors specifically in these key players in PGE_2_ pathway have compromised the interpretation of those associations. Furthermore, we used the data-mining analytical approach, MDR, to enhance the likelihood of identifying gene-gene interactions and a strong interaction between four SNPs in *HPGD*, *SCO2A1* and *ABCC4* genes reinforcing the data from single–locus analysis and lending further support to the involvement of genetic susceptibility biomarkers in colorectal carcinogenesis.

There are a few limitations that should be considered. First, this study has a case-control design, so we could not rule out selection bias, although if this was the case our results would tend to have strong associations; or recall bias that could decrease the accuracy of collected data. Second, our sample size allowed us to detect strong associations in the overall analysis for frequent polymorphisms, so we cannot exclude the influence of rarer SNPs or with more modest influences in the *PTGS2/HPGD/SLCO2A1/ABCC4* genes in CRC development. Furthermore, and although we employed statistical strategies to assess the robustness of associations, namely the use of bootstrap resampling, an independent and larger data set is needed to corroborate our findings and allow a more comprehensive understanding of the gene-environment interactions.

In conclusion, we observed that seven tagSNPs in key genes regulating the procarcinogenic-PGE_2_ levels in tumor microenvironment were implicated in CRC development. Particularly, the *COX-2* rs689466GG genotype in ever-smokers and a gene-gene interaction involving the rs1426945 *HPGD* polymorphism, rs6439448 *SLCO2A1* SNP and rs1751051 and rs1751031 polymorphisms in *ABCC4* gene. A clearer understanding on CRC etiology through the identification of risk biomarkers might allow a better definition of risk models that are more likely to benefit from targeted preventive strategies.

## Supporting Information

Table S1 Genetic polymorphisms in *COX-2/HPGD/SLCO2A1/ABCC4* genes characterization and quality control results.
(DOCX)

Table S2 Genotype frequencies among cases and controls and risk estimates for the involvement of *COX-2/HPGD/SLCO2A1/ABCC4* polymorphisms in colorectal cancer onset.
(DOCX)
