# Supplementary material for: Genetic Variability in Key Genes in Prostaglandin E2 Pathway (COX-2, HPGD, ABCC4 and SLCO2A1) and Their Involvement in Colorectal Cancer Development
Source: PLoS One. 2014 Apr 2;9(4):e92000. doi: 10.1371/journal.pone.0092000 (PMC3973663; doi:10.1371/journal.pone.0092000)
Supplement: Table S1 — Genetic polymorphisms in COX-2/HPGD/SLCO2A1/ABCC4 genes characterization and quality control results. (DOCX) [file pone.0092000.s001.docx]

| Table S1. Genetic polymorphisms in *COX-2/HPGD/SLCO2A1/ABCC4* genes characterization and quality control results | | | | | | |
| --- | --- | --- | --- | --- | --- | --- |
| Gene | tagSNP | Other SNPs on the block | Genotype call rate | Genotype concordance rate | HWE | Passed quality check? |
| *COX-2* | rs689466 | Candidate gene | 98.5 | 0.97 | 0.901 | Yes |
|  | rs20417 | Candidate gene | 98.1 | 0.96 | 0.998 | Yes |
|  | rs5275 | Candidate gene | 96.7 | 1.00 | 0.999 | Yes |
| *HPGD* | rs2555639 | Candidate gene | 98.4 | 1.00 | 0.989 | Yes |
|  | rs1346271 | singleton | 99.6 | 1.00 | 0.167 | Yes |
|  | rs2555632 | rs3101255 | 99.6 | 1.00 | 0.681 | Yes |
|  | rs2303520 | rs13127058 | 99.2 | 1.00 | 0.633 | Yes |
|  | rs1863642 | rs2612659 | 99.2 | 1.00 | 0.474 | Yes |
|  | rs1426945 | rs3756273 | 99.6 | 0.97 | 0.976 | Yes |
|  | rs12500316 | rs1863641 | 99.2 | 1.00 | 0.508 | Yes |
|  |  | rs11722919 |  |  |  |  |
|  | rs8752 | rs1426947 | 99.6 | 1.00 | 0.948 | Yes |
|  |  | rs2612658 |  |  |  |  |
|  |  | rs11133041 |  |  |  |  |
|  |  | rs11724251 |  |  |  |  |
|  | rs2612656 | rs1816204 | 92.2 | 0.96 | 0.917 | Yes |
|  |  | rs3857075 |  |  |  |  |
| *SLCO2A1* | rs4241362 | rs4241361 | 98.8 | 1.00 | 0.756 | Yes |
|  |  | rs4634113 |  |  |  |  |
|  |  | rs6804798 |  |  |  |  |
|  |  | rs9828294 |  |  |  |  |
|  |  | rs9855403 |  |  |  |  |
|  |  | rs9874493 |  |  |  |  |
|  |  | rs9882333 |  |  |  |  |
|  |  | rs11720811 |  |  |  |  |
|  | rs764392 | rs4327389 | 98.5 | 1.00 | 0.550 | Yes |
|  |  | rs4854777 |  |  |  |  |
|  |  | rs5013525 |  |  |  |  |
|  |  | rs7646298 |  |  |  |  |
|  |  | rs7646473 |  |  |  |  |
|  |  | rs12695600 |  |  |  |  |
|  | rs6439448 | rs2370512 | 99.0 | 0.97 | 0.979 | Yes |
|  |  | rs3923835 |  |  |  |  |
|  |  | rs3923835 |  |  |  |  |
|  |  | rs4854768 |  |  |  |  |
|  |  | rs4854769 |  |  |  |  |
|  |  | rs34550074 |  |  |  |  |
|  | rs9821091 | rs7630191 | 99.7 | 1.00 | 0.651 | Yes |
|  |  | rs9841380 |  |  |  |  |
|  |  | rs6439450 |  |  |  |  |
|  |  | rs7617777 |  |  |  |  |
|  |  | rs9834727 |  |  |  |  |
|  | rs9820625 | rs9836830 | 99.6 | 1.00 | 0.948 | Yes |
|  |  | rs9917636 |  |  |  |  |
|  |  | rs11709172 |  |  |  |  |
|  |  | rs13083175 |  |  |  |  |
|  | rs9834412 | rs4854785 | 98.4 | 1.00 | 0.951 | Yes |
|  |  | rs13067921 |  |  |  |  |
|  | rs4241365 | rs7653639 | 99.0 | 1.00 | 0.923 | Yes |
|  | rs4331673 | rs11720843 | 99.6 | 1.00 | 0.994 | Yes |
|  | rs4854784 | rs7636169 | 98.1 | 0.97 | 0.871 | Yes |
|  | rs7340717 | rs7340718 | 98.4 | 1.00 | 0.600 | Yes |
|  | rs7616492 | rs10935089 | 98.9 | 1.00 | 0.938 | Yes |
|  | rs7625035 | rs9822027 | 99.5 | 1.00 | 0.455 | Yes |
|  | rs1131598 | Singleton | 99.5 | 1.00 | 0.441 | Yes |
|  | rs10935090 | Singleton | 99.0 | 1.00 | 0.815 | Yes |
|  | rs11915399 | Singleton | 99.7 | 1.00 | 0.998 | Yes |
| *ABCC4* | rs4148422 | rs17300935 | 99.6 | 0.95 | 0.006 | No |
|  | rs9524821 | [rs9516532] | 99.2 | 1.00 | 0.604 | Yes |
|  | rs3782958 | rs4148515 | 99.3 | 1.00 | 0.931 | Yes |
|  |  | rs10508023 |  |  |  |  |
|  | rs869951 | rs871052 | 99.7 | 1.00 | 0.854 | Yes |
|  |  | rs8001444 |  |  |  |  |
|  | rs4771912 | rs7981095 | 98.8 | 0.97 | 0.936 | Yes |
|  | rs4148421 | rs9524864 | 98.2 | 1.00 | 0.998 | Yes |
|  |  | rs9524873 |  |  |  |  |
|  |  | rs10508017 |  |  |  |  |
|  | rs8002180 | rs4148424 | 99.5 | 1.00 | 0.694 | Yes |
|  |  | rs4771910 |  |  |  |  |
|  |  | rs7317112 |  |  |  |  |
|  |  | rs7322318 |  |  |  |  |
|  |  | rs8001475 |  |  |  |  |
|  |  | rs9584288 |  |  |  |  |
|  |  | rs9590228 |  |  |  |  |
|  | rs9590222 | rs12100301 | 94.6 | 1.00 | <0.001 | No |
|  | rs2127295 | rs2698243 | 99.0 | 0.97 | 0.657 | Yes |
|  |  | rs1564355 |  |  |  |  |
|  |  | rs1617785 |  |  |  |  |
|  |  | rs1630807 |  |  |  |  |
|  |  | rs1678363 |  |  |  |  |
|  |  | rs1678394 |  |  |  |  |
|  |  | rs1729748 |  |  |  |  |
|  |  | rs2766481 |  |  |  |  |
|  |  | rs3825415 |  |  |  |  |
|  |  | rs6650282 |  |  |  |  |
|  | rs1751051 | [rs1751050] | 99.0 | 1.00 | 0.999 | Yes |
|  | rs9590220 | rs9590216 | 99.5 | 0.96 | 0.018 | No |
|  |  | rs17235152 |  |  |  |  |
|  | rs2892715 | rs9561814 | 99.7 | 1.00 | 0.473 | Yes |
|  | rs2892713 | rs12865305 | 99.6 | 1.00 | 0.313 | Yes |
|  | rs4612933 | rs899494 | 99.5 | 1.00 | 0.936 | Yes |
|  |  | rs899495 |  |  |  |  |
|  |  | rs899496 |  |  |  |  |
|  |  | rs1678403 |  |  |  |  |
|  |  | rs1824911 |  |  |  |  |
|  |  | rs1824913 |  |  |  |  |
|  |  | rs1926657 |  |  |  |  |
|  |  | rs3782965 |  |  |  |  |
|  |  | rs4148465 |  |  |  |  |
|  |  | rs4148469 |  |  |  |  |
|  |  | rs4303338 |  |  |  |  |
|  |  | rs4334136 |  |  |  |  |
|  |  | rs4505186 |  |  |  |  |
|  |  | rs4773854 |  |  |  |  |
|  |  | rs4773855 |  |  |  |  |
|  |  | rs7325019 |  |  |  |  |
|  |  | rs7333234 |  |  |  |  |
|  |  | rs7335147 |  |  |  |  |
|  |  | rs7983336 |  |  |  |  |
|  |  | rs7987653 |  |  |  |  |
|  |  | rs7988494 |  |  |  |  |
|  |  | rs9524831 |  |  |  |  |
|  |  | rs9524833 |  |  |  |  |
|  |  | rs9524845 |  |  |  |  |
|  |  | rs9524856 |  |  |  |  |
|  |  | rs12870204 |  |  |  |  |
|  | rs4148437 | rs9556466 | 99.3 | 1.00 | 0.665 | Yes |
|  |  | rs2892716 |  |  |  |  |
|  |  | rs4148436 |  |  |  |  |
|  |  | rs4148446 |  |  |  |  |
|  |  | rs10508018 |  |  |  |  |
|  | rs12867485 | rs9561811 | 0 | - | - | No |
|  |  | rs17189481 |  |  |  |  |
|  | rs1611822 | rs1751015 | 99.6 | 1.00 | 0.969 | Yes |
|  | rs1678386 | rs9516530 | 99.6 | 1.00 | 0.818 | Yes |
|  | rs2274403 | rs3864997 | 99.6 | 0.97 | 0.862 | Yes |
|  |  | rs4148481 |  |  |  |  |
|  | rs17268122 | rs17268163 | 92.6 | 0.96 | 0.027 | No |
|  | rs1751027 | rs1564351 | 99.7 | 1.00 | 0.396 | Yes |
|  |  | rs4148487 |  |  |  |  |
|  |  | rs17189390 |  |  |  |  |
|  |  | rs17268170 |  |  |  |  |
|  | rs4148476 | rs4773843 | 99.5 | 1.00 | 0.280 | Yes |
|  |  | rs9524822 |  |  |  |  |
|  | rs1678374 | rs1751025 | 99.3 | 1.00 | 0.964 | Yes |
|  | rs1678405 | rs2793821 | 98.9 | 1.00 | 0.722 | Yes |
|  |  | rs6492768 |  |  |  |  |
|  |  | rs7330933 |  |  |  |  |
|  | rs1678396 | rs2766482 | 99.7 | 1.00 | 0.537 | Yes |
|  | rs1628382 | rs4148527 | 26.5 | 1.00 | - | No |
|  |  | rs8001657 |  |  |  |  |
|  |  | rs12584534 |  |  |  |  |
|  | rs1678354 | rs1751059 | 0 | - | - | No |
|  | rs1751031 | rs931111 | 99.7 | 1.00 | 0.372 | Yes |
|  |  | rs1189444 |  |  |  |  |
|  |  | rs1189451 |  |  |  |  |
|  |  | rs1189452 |  |  |  |  |
|  |  | rs1729747 |  |  |  |  |
|  |  | rs2619312 |  |  |  |  |
|  |  | rs5016378 |  |  |  |  |
|  | rs7993878 | rs9302040 | 99.9 | 0.97 | 0.346 | Yes |
|  |  | rs9302042 |  |  |  |  |
|  |  | rs9302043 |  |  |  |  |
|  |  | rs9556455 |  |  |  |  |
|  |  | rs9561768 |  |  |  |  |
|  |  | rs9561769 |  |  |  |  |
|  |  | rs9590168 |  |  |  |  |
|  |  | rs10219913 |  |  |  |  |
|  | rs6492763 | rs10508024 | 98.8 | 0.97 | 0.520 | Yes |
|  | rs3742106 | rs4148544 | 99.3 | 1.00 | 0.987 | Yes |
|  |  | rs4148549 |  |  |  |  |
|  |  | rs4148551 |  |  |  |  |
|  |  | rs7330196 |  |  |  |  |
|  |  | rs9302039 |  |  |  |  |
|  |  | rs9524769 |  |  |  |  |
