# Supplementary material for: Genetic Variability in Key Genes in Prostaglandin E2 Pathway (COX-2, HPGD, ABCC4 and SLCO2A1) and Their Involvement in Colorectal Cancer Development
Source: PLoS One. 2014 Apr 2;9(4):e92000. doi: 10.1371/journal.pone.0092000 (PMC3973663; doi:10.1371/journal.pone.0092000)
Supplement: Table S2 — Genotype frequencies among cases and controls and risk estimates for the involvement of COX-2/HPGD/SLCO2A1/ABCC4 polymorphisms in colorectal cancer onset. (DOCX) [file pone.0092000.s002.docx]

| Table S2. Genotype frequencies among cases and controls and risk estimates for the involvement of *COX-2/HPGD/SLCO2A1/ABCC4* polymorphisms in colorectal cancer onset | | | | | | | | |
| --- | --- | --- | --- | --- | --- | --- | --- | --- |
| SNPs rs | Cases  n (%) | Controls  n (%) | OR | 95% CI | *P* value | OR | 95% CI | *aP* value |
| *COX-2* |  |  |  |  |  |  |  |  |
| rs689466 |  |  |  |  |  |  |  |  |
| AA | 143 (58.8) | 323 (68.4) | 1.00 | Reference | - | 1.00 | Reference | - |
| AG | 85 (35.0) | 133 (28.2) | 1.44 | 1.03-2.02 | **0.032** | 1.53 | 1.08-2.17 | 0.022 |
| GG | 15 (6.2) | 16 (3.4) | 2.12 | 1.02-4.40 | **0.040** | 2.02 | 0.93-4.39 |  |
| rs20417 |  |  |  |  |  |  |  |  |
| GG | 179 (74.9) | 328 (69.3) | 1.00 | Reference | - | 1.00 | Reference | - |
| GC | 55 (23.0) | 132 (27.9) | 0.76 | 0.53-1.10 | 0.145 | 0.80 | 0.55-1.16 | 0.390 |
| CC | 5 (2.1) | 13 (2.7) | 0.71 | 0.25-2.01 | 0.511 | 0,64 | 0.22-1.90 |  |
| s5275 |  |  |  |  |  |  |  |  |
| TT | 122 (50.8) | 235 (50.9) | 1.00 | Reference | - | 1.00 | Reference | - |
| TC | 89 (37.1) | 189 (40.9) | 0.91 | 0.65-1.27 | 0.567 | 0.93 | 0.66-1.32 | 0.390 |
| CC | 29 (12.1) | 38 (8.2) | 1.47 | 0.86-2.50 | 0.153 | 1.39 | 0.80-2.41 |  |
| *HPGD* |  |  |  |  |  |  |  |  |
| rs2555629 |  |  |  |  |  |  |  |  |
| TT | 111 (46.1) | 216 (45.6) | 1.00 | Reference | - | 1.00 | Reference | - |
| TC | 101 (41.9) | 209 (44.1) | 0.94 | 0.68-1.31 | 0.715 | 0.99 | 0.70-1.40 | 0.960 |
| CC | 29 (12.0) | 49 (10.3) | 1.15 | 0.69-1.92 | 0.589 | 1.07 | 0.63-1.83 |  |
| rs2612656 |  |  |  |  |  |  |  |  |
| AA | 160 (72.7) | 295 (65.6) | 1.00 | Reference | - | 1.00 | Reference | - |
| AG | 47 (21.4) | 137 (30.4) | 0.63 | 0.43-0.93 | **0.019** | 0.71 | 0.48-1.05 | 0.110 |
| GG | 13 (5.9) | 18 (4.0) | 1.33 | 0.64-2.79 | 0.446 | 1.48 | 0.69-3.18 |  |
| rs8752 |  |  |  |  |  |  |  |  |
| TT | 91 (37.1) | 197 (41.2) | 1.00 | reference | - | 1.00 | Reference | - |
| TC | 112 (45.7) | 219 (45.8) | 1.11 | 0.79-1.55 | 0.553 | 1.16 | 0.82-1.65 | 0.398 |
| CC | 42 (17.1) | 62 (13.0) | 1.47 | 0.92-2.33 | 0.105 | 1.61 | 0.98-2.62 | 0.059 |
| rs1346271 |  |  |  |  |  |  |  |  |
| GG | 104 (42.4) | 174 (36.2) | 1.00 | reference | - | 1.00 | Reference | - |
| GC | 97 (39.6) | 246 (51.2) | 0.66 | 0.47-0.92 | **0.016** | **0.68** | **0.47-0.96** | **0.029** |
| CC | 44 (18.0) | 60 (12.5) | 1.23 | 0.78-1.94 | 0.382 | 1.34 | 0.83-2.17 | 0.231 |
| rs2555632 |  |  |  |  |  |  |  |  |
| TT | 143 (58.4) | 284 (59.3) | 1.00 | reference | - | 1.00 | Reference | - |
| TC | 88 (35.9) | 174 (36.3) | 1.00 | 0.72-1.39 | 0.979 | 1.14 | 0.81-1.60 | 0.470 |
| CC | 14 (5.7) | 21 (4.4) | 1.32 | 0.65-2.68 | 0.434 | 1.44 | 0.69-3.00 | 0.331 |
| rs2303520 |  |  |  |  |  |  |  |  |
| GG | 167 (69.3) | 342 (71.4) | 1.00 | reference | - | 1.00 | Reference | - |
| GA | 66 (27.4) | 123 (25.7) | 1.10 | 0.77-1.56 | 0.599 | 1.09 | 0.76-1.58 | 0.641 |
| AA | 8 (3.3) | 14 (2.9) | 1.17 | 0.48-2.84 | 0.728 | 1.30 | 0.52-3.28 | 0.576 |
| rs1863642 |  |  |  |  |  |  |  |  |
| GG | 126 (52.3) | 231 (48.1) | 1.00 | reference | - | 1.00 | Reference | - |
| GT | 96 (39.8) | 212 (44.2) | 0.83 | 0.60-1.15 | 0.261 | 0.81 | 0.58-1.14 | 0.233 |
| TT | 19 (7.9) | 37 (7.7) | 0.94 | 0.52-1.71 | 0.842 | 0.94 | 0.51-1.76 | 0.854 |
| rs1426945 |  |  |  |  |  |  |  |  |
| GG | 110 (44.7) | 169 (35.3) | 1.00 | reference | - | 1.00 | Reference | - |
| GA | 108 (43.9) | 233 (48.6) | 0.71 | 0.51-0.99 | **0.044** | 0.70 | 0.50-1.00 | 0.050 |
| AA | 28 (11.4) | 77 (16.1) | 0.56 | 0.34-0.92 | **0.020** | **0.56** | **0.34-0.93** | **0.026** |
| rs12500316 |  |  |  |  |  |  |  |  |
| CC | 150 (62.0) | 262 (54.7) | 1.00 | reference | - | 1.00 | Reference | - |
| CT | 78 (32.2) | 191 (39.9) | 0.71 | 0.51-0.99 | **0.045** | 0.73 | 0.52-1.03 | 0.071 |
| TT | 14 (5.8) | 26 (5.4) | 0.94 | 0.48-1.86 | 0.860 | 1.01 | 0.50-2.08 | 0.972 |
| *SLCO2A1* |  |  |  |  |  |  |  |  |
| rs4241362 |  |  |  |  |  |  |  |  |
| TT | 152 (63.3) | 333 (69.5) | 1.00 | reference | - | 1.00 | Reference | - |
| TC | 73 (30.4) | 130 (27.1) | 1.23 | 0.87-1.74 | 0.239 | 1.14 | 0.79-1.63 | 0.487 |
| CC | 15 (6.2) | 16 (3.3) | 2.05 | 0.99-4.26 | **0.049** | 1.82 | 0.84-3.94 | 0.130 |
| rs7646392 |  |  |  |  |  |  |  |  |
| CC | 92 (38.8) | 175 (36.5) | 1.00 | reference | - | 1.00 | Reference | - |
| CT | 97 (40.9) | 220 (45.8) | 0.84 | 0.59-1.19 | 0.321 | 0.97 | 0.67-1.40 | 0.869 |
| TT | 48 (20.3) | 85 (17.7) | 1.07 | 0.70-1.66 | 0.747 | 1.28 | 0.81-2.04 | 0.286 |
| rs6439448 |  |  |  |  |  |  |  |  |
| CC | 174 (72.2) | 320 (66.7) | 1.00 | reference | - | 1.00 | Reference | - |
| CA | 56 (23.2) | 143 (29.8) | 0.72 | 0.50-1.03 | 0.073 | **0.68** | **0.47-1.00** | **0.047** |
| AA | 11 (4.6) | 17 (3.5) | 1.19 | 0.54-2.60 | 0.662 | 0.93 | 0.39-2.20 | 0.869 |
| rs9821091 |  |  |  |  |  |  |  |  |
| GG | 110 (44.5) | 180 (37.6) | 1.00 | reference | - | 1.00 | Reference | - |
| GA | 105 (42.5) | 235 (49.1) | 0.73 | 0.52-1.02 | 0.063 | 0.79 | 0.56-1.12 | 0.181 |
| AA | 32 (13.0) | 64 (13.4) | 0.82 | 0.50-1.33 | 0.418 | 0.86 | 0.52-1.43 | 0.561 |
| rs9820625 |  |  |  |  |  |  |  |  |
| AA | 77 (31.3) | 141 (29.4) | 1.00 | reference | - | 1.00 | Reference | - |
| AC | 110 (44.7) | 232 (48.3) | 0.87 | 0.61-1.24 | 0.440 | 1.00 | 0.69-1.46 | 0.998 |
| CC | 59 (24.0) | 107 (22.3) | 1.01 | 0.66-1.54 | 0.964 | 1.14 | 0.73-1.78 | 0.574 |
| rs9834412 |  |  |  |  |  |  |  |  |
| CC | 140 (59.6) | 270 (56.1) | 1.00 | reference | - | 1.00 | Reference | - |
| CA | 76 (32.3) | 179 (37.2) | 0.82 | 0.58-1.15 | 0.245 | 0.78 | 0.55-1.11 | 0.170 |
| AA | 19 (8.1) | 32 (6.7) | 1.14 | 0.63-2.09 | 0.660 | 1.13 | 0.60-2.11 | 0.707 |
| rs4241365 |  |  |  |  |  |  |  |  |
| TT | 156 (64.5) | 282 (58.9) | 1.00 | reference | - | 1.00 | Reference | - |
| TC | 72 (29.8) | 169 (35.3) | 0.77 | 0.55-1.08 | 0.130 | 0.82 | 0.57-1.16 | 0.255 |
| CC | 14 (5.8) | 28 (5.8) | 0.90 | 0.46-1.77 | 0.768 | 1.05 | 0.52-2.13 | 0.888 |
| rs4331673 |  |  |  |  |  |  |  |  |
| CC | 153 (62.4) | 332 (69.2) | 1.00 | reference | - | 1.00 | Reference | - |
| CA | 84 (34.3) | 134 (27.9) | 1.36 | 0.98-1.90 | 0.070 | 1.30 | 0.92-1.84 | 0.145 |
| AA | 8 (3.3) | 14 (2.9) | 1.24 | 0.51-3.02 | 0.635 | 1.22 | 0.48-3.06 | 0.679 |
| rs4854784 |  |  |  |  |  |  |  |  |
| GG | 106 (45.1) | 215 (44.9) | 1.00 | reference | - | 1.00 | Reference | - |
| GA | 97 (41.3) | 208 (43.4) | 0.95 | 0.68-1.32 | 0.745 | 1.00 | 0.70-1.41 | 0.989 |
| AA | 32 (13.6) | 56 (11.7) | 1.16 | 0.71-1.90 | 0.557 | 1.35 | 0.80-2.28 | 0.255 |
| rs7340717 |  |  |  |  |  |  |  |  |
| GG | 105 (44.5) | 204 (42.5) | 1.00 | reference | - | 1.00 | Reference | - |
| GT | 90 (38.1) | 210 (43.8) | 0.83 | 0.59-1.17 | 0.293 | 0.83 | 0.58-1.18 | 0.299 |
| TT | 41 (17.4) | 66 (13.8) | 1.21 | 0.76-1.90 | 0.418 | 0.99 | 0.61-1.60 | 0.952 |
| rs7616492 |  |  |  |  |  |  |  |  |
| GG | 89 (37.1) | 202 (42.1) | 1.00 | reference | - | 1.00 | Reference | - |
| GA | 103 (42.9) | 216 (45.0) | 1.08 | 0.77-1.52 | 0.651 | 1.18 | 0.82-1.69 | 0.373 |
| AA | 48 (20.0) | 62 (12.9) | 1.76 | 1.12-2.76 | **0.014** | **2.05** | **1.27-3.32** | **0.003** |
| rs7625035 |  |  |  |  |  |  |  |  |
| AA | 139 (57.0) | 278 (57.9) | 1.00 | reference | - | 1.00 | Reference | - |
| AG | 85 (34.8) | 181 (37.7) | 0.94 | 0.68-1.30 | 0.708 | 0.92 | 0.65-1.29 | 0.619 |
| GG | 20 (8.2) | 21 (4.4) | 1.91 | 1.00-3.63 | **0.047** | 1.60 | 0.82-3.12 | 0.168 |
| rs1131598 |  |  |  |  |  |  |  |  |
| AA | 136 (55.7) | 274 (5.1) | 1.00 | reference | - | 1.00 | Reference | - |
| AG | 88 (36.1) | 184 (38.3) | 0.96 | 0.70-1.34 | 0.824 | 0.90 | 0.64-1.27 | 0.563 |
| GG | 20 (8.2) | 22 (4.6) | 1.83 | 0.97-3.47 | 0.061 | 1.79 | 0.92-3.50 | 0.087 |
| rs10935090 |  |  |  |  |  |  |  |  |
| CC | 180 (74.4) | 382 (79.7) | 1.00 | reference | - | 1.00 | Reference | - |
| CT | 54 (22.3) | 90 (18.8) | 1.27 | 0.87-1.86 | 0.213 | 1.28 | 0.86-1.90 | 0.222 |
| TT | 8 (3.3) | 7 (1.5) | 2.42 | 0.87-6.79 | 0.082 | 2.33 | 0.80-6.83 | 0.123 |
| rs11915399 |  |  |  |  |  |  |  |  |
| CC | 173 (70.0) | 328 (68.5) | 1.00 | reference | - | 1.00 | Reference | - |
| CT | 66 (26.7) | 137 (28.6) | 0.91 | 0.65-1.29 | 0.608 | 0.99 | 0.69-1.42 | 0.953 |
| TT | 8 (3.2) | 14 (2.9) | 1.08 | 0.45-2.63 | 0.860 | 1.11 | 0.44-2.79 | 0.832 |
| ABCC4 |  |  |  |  |  |  |  |  |
| rs9524821 |  |  |  |  |  |  |  |  |
| GG | 92 (37.9) | 205 (42.8) | 1.00 | reference | - | 1.00 | Reference | - |
| GA | 116 (47.7) | 209 (43.6) | 1.24 | 0.88-1.73 | 0.213 | 1.30 | 0.91-1.84 | 0.145 |
| AA | 35 (14.4) | 65 (13.6) | 1.20 | 0.74-1.94 | 0.456 | 1.18 | 0.70-1.98 | 0.534 |
| rs3782958 |  |  |  |  |  |  |  |  |
| GG | 173 (70.9) | 336 (70.1) | 1.00 | reference | - | 1.00 | Reference | - |
| GC | 62 (25.4) | 129 (26.9) | 0.93 | 0.66-1.33 | 0.700 | 0.89 | 0.62-1.29 | 0.894 |
| CC | 9 (3.7) | 14 (2.9) | 1.25 | 0.53-2.94 | 0.611 | 1.15 | 0.48-2.78 | 0.755 |
| rs869951 |  |  |  |  |  |  |  |  |
| GG | 101 (41.1) | 171 (35.6) | 1.00 | reference | - | 1.00 | Reference | - |
| GC | 103 (41.9) | 226 (47.1) | 0.77 | 0.55-1.08 | 0.133 | 0.75 | 0.53-1.07 | 0.120 |
| CC | 42 (17.1) | 83 (17.3) | 0.86 | 0.55-1.34 | 0.496 | 0.88 | 0.56-1.40 | 0.600 |
| rs4771912 |  |  |  |  |  |  |  |  |
| AA | 189 (79.4) | 359 (74.6) | 1.00 | reference | - | 1.00 | Reference | - |
| AG | 48 (20.2) | 112 (23.3) | 0.81 | 0.56-1.19 | 0.290 | 0.80 | 0.54-1.19 | 0.275 |
| GG | 1 (0.4) | 10 (2.1) | 0.19 | 0.02-1.50 | 0.078 | 0.17 | 0.02-1.38 | 0.097 |
| rs4148421 |  |  |  |  |  |  |  |  |
| GG | 71 (30.1) | 134 (28.0) | 1.00 | reference | - | 1.00 | Reference | - |
| GA | 111 (47.0) | 238 (49.7) | 0.88 | 0.61-1.27 | 0.494 | 0.84 | 0.57-1.24 | 0.381 |
| AA | 54 (22.9) | 107 (22.3) | 0.95 | 0.62-1.47 | 0.827 | 1.02 | 0.64-1.61 | 0.939 |
| rs8002180 |  |  |  |  |  |  |  |  |
| TT | 124 (50.6) | 248 (51.8) | 1.00 | reference | - | 1.00 | Reference | - |
| TC | 97 (39.6) | 188 (39.2) | 1.03 | 0.74-1.43 | 0.850 | 0.98 | 0.70-1.38 | 0.909 |
| CC | 24 (9.8) | 43 (9.0) | 1.12 | 0.65-1.92 | 0.692 | 1.03 | 0.58-1.84 | 0.913 |
| rs2127295 |  |  |  |  |  |  |  |  |
| GG | 70 (28.8) | 137 (28.7) | 1.00 | reference | - | 1.00 | Reference | - |
| GA | 130 (53.5) | 247 (51.7) | 1.03 | 0.72-1.47 | 0.871 | 1.00 | 0.69-1.45 | 0.998 |
| AA | 43 (17.7) | 94 (19.7) | 0.90 | 0.56-1.42 | 0.639 | 0.85 | 0.52-1.38 | 0.508 |
| rs1751051 |  |  |  |  |  |  |  |  |
| TT | 112 (46.5) | 234 (48.8) | 1.00 | reference | - | 1.00 | Reference | - |
| TA | 91 (37.8) | 202 (42.1) | 0.94 | 0.67-1.32 | 0.723 | 1.06 | 0.74-1.50 | 0.764 |
| AA | 38 (15.8) | 44 (9.2) | 1.80 | 1.11-2.94 | **0.017** | **1.76** | **1.04-2.95** | **0.034** |
| rs2892715 |  |  |  |  |  |  |  |  |
| GG | 103 (42.0) | 173 (36.0) | 1.00 | reference | - | 1.00 | Reference | - |
| GA | 100 (40.8) | 220 (45.7) | 0.76 | 0.54-1.07 | 0.119 | 0.74 | 0.52-1.06 | 0.102 |
| AA | 42 (17.1) | 88 (18.3) | 0.80 | 0.52-1.25 | 0.326 | 0.82 | 0.52-1.30 | 0.406 |
| rs2892713 |  |  |  |  |  |  |  |  |
| CC | 169 (68.7) | 337 (70.4) | 1.00 | reference | - | 1.00 | Reference | - |
| CT | 66 (26.8) | 124 (25.9) | 1.06 | 0.75-1.51 | 0.740 | 1.13 | 0.78-1.63 | 0.514 |
| TT | 11 (4.5) | 18 (3.8) | 1.22 | 0.56-2.64 | 0.615 | 1.25 | 0.56-2.81 | 0.582 |
| rs4612933 |  |  |  |  |  |  |  |  |
| CC | 160 (65.8) | 315 (65.5) | 1.00 | reference | - | 1.00 | Reference | - |
| CT | 71 (29.2) | 148 (30.8) | 0.94 | 0.67-1.33 | 0.743 | 0.95 | 0.66-1.36 | 0.777 |
| TT | 12 (4.9) | 18 (3.7) | 1.31 | 0.62-2.79 | 0.479 | 1.31 | 0.59-2.90 | 0.510 |
| rs4148437 |  |  |  |  |  |  |  |  |
| TT | 107 (44.0) | 194 (40.4) | 1.00 | reference | - | 1.00 | Reference | - |
| TC | 104 (42.8) | 215 (44.8) | 0.88 | 0.63-1.22 | 0.439 | 0.80 | 0.56-1-13 | 0.201 |
| CC | 32 (13.2) | 71 (14.8) | 0.82 | 0.51-1.32 | 0.409 | 0.81 | 0.50-1.33 | 0.406 |
| rs1611822 |  |  |  |  |  |  |  |  |
| CC | 77 (31.4) | 182 (37.9) | 1.00 | reference | - | 1.00 | Reference | - |
| CT | 126 (51.4) | 225 (46.9) | 1.32 | 0.94-1.87 | 0.110 | 1.44 | 0.89-2.07 | 0.060 |
| TT | 42 (17.1) | 73 (15.2) | 1.36 | 0.86-2.16 | 0.193 | 1.38 | 0.85-2.22 | 0.190 |
| rs1678386 |  |  |  |  |  |  |  |  |
| AA | 124 (50.6) | 243 (50.6) | 1.00 | reference | - | 1.00 | Reference | - |
| AC | 90 (36.7) | 193 (40.2) | 0.91 | 0.66-1.27 | 0.593 | 0.90 | 0.64-1.27 | 0.541 |
| CC | 31 (12.7) | 44 (9.2) | 1.38 | 0.83-2.30 | 0.212 | 1.33 | 0.77-2.30 | 0.304 |
| rs2274403 |  |  |  |  |  |  |  |  |
| AA | 74 (30.2) | 120 (25.0) | 1.00 | reference | - | 1.00 | Reference | - |
| AG | 122 (49.8) | 234 (48.8) | 0.84 | 0.59-1.22 | 0.365 | 0.90 | 0.61-1.32 | 0.594 |
| GG | 49 (20.0) | 126 (26.2) | 0.63 | 0.41-0.98 | **0.039** | 0.66 | 0.42-1.03 | 0.067 |
| rs1751027 |  |  |  |  |  |  |  |  |
| AA | 202 (82.1) | 402 (83.8) | 1.00 | reference | - | 1.00 | Reference | - |
| AG | 40 (16.3) | 77 (16.0) | 1.03 | 0.68-1.57 | 0.876 | 1.13 | 0.73-1.75 | 0.588 |
| GG | 4 (1.6) | 1 (0.2) | 7.96 | 0.88-71.69 | **0.047** | 6.11 | 0.65-57.40 | 0.113 |
| rs4148476 |  |  |  |  |  |  |  |  |
| TT | 181 (74.2) | 339 (70.6) | 1.00 | reference | - | 1.00 | Reference | - |
| TG | 56 (23.0) | 123 (25.6) | 0.85 | 0.59-1.23 | 0.390 | 0.89 | 0.61-1.31 | 0.559 |
| GG | 7 (2.9) | 18 (3.8) | 0.73 | 0.30-1.78 | 0.484 | 0.76 | 0.30-1.92 | 0.565 |
| rs1678374 |  |  |  |  |  |  |  |  |
| TT | 90 (37.0) | 164 (34.2) | 1.00 | reference | - | 1.00 | Reference | - |
| TC | 118 (48.6) | 235 (49.1) | 0.92 | 0.65-1.28 | 0.608 | 1.02 | 0.71-1.45 | 0.931 |
| CC | 35 (14.4) | 80 (16.7) | 0.80 | 0.50-1.28 | 0.347 | 0.77 | 0.48-1.26 | 0.303 |
| rs1678405 |  |  |  |  |  |  |  |  |
| TT | 116 (48.5) | 199 (41.4) | 1.00 | reference | - | 1.00 | Reference | - |
| TC | 103 (43.1) | 227 (47.2) | 0.78 | 0.56-1.08 | 0.132 | 0.75 | 0.54-1.06 | 0.104 |
| CC | 20 (8.4) | 55 (11.4) | 0.62 | 0.36-1.09 | 0.097 | 0.56 | 0.31-1.01 | 0.056 |
| rs1678396 |  |  |  |  |  |  |  |  |
| TT | 94 (38.2) | 147 (30.6) | 1.00 | reference | - | 1.00 | Reference | - |
| TC | 105 (42.7) | 248 (51.7) | 0.66 | 0.47-0.94 | **0.019** | 0.73 | 0.50-1.04 | 0.084 |
| CC | 47 (19.1) | 85 (17.7) | 0.86 | 0.56-1.34 | 0.518 | 0.88 | 0.56-1.40 | 0.590 |
| rs1751031 |  |  |  |  |  |  |  |  |
| AA | 165 (67.1) | 299 (62.3) | 1.00 | reference | - | 1.00 | Reference | - |
| AG | 66 (26.8) | 166 (34.6) | 0.72 | 0.51-1.02 | 0.060 | **0.68** | **0.47-0.97** | **0.032** |
| GG | 15 (6.1) | 15 (3.1) | 1.81 | 0.86-3.80 | 0.111 | 1.67 | 0.77-3.63 | 0.194 |
| rs7993878 |  |  |  |  |  |  |  |  |
| GG | 190 (77.2) | 361 (75.1) | 1.00 | reference | - | 1.00 | Reference | - |
| GA | 47 (19.1) | 107 (22.2) | 0.84 | 0.57-1.23 | 0.357 | 0.77 | 0.52-1.15 | 0.204 |
| AA | 9 (3.7) | 13 (2.7) | 1.32 | 0.55-3.13 | 0.535 | 1.26 | 0.49-3.23 | 0.634 |
| rs6492763 |  |  |  |  |  |  |  |  |
| TT | 89 (37.2) | 168 (35.0) | 1.00 | reference | - | 1.00 | Reference | - |
| TC | 108 (45.2) | 242 (50.4) | 0.84 | 0.60-1.19 | 0.327 | 0.80 | 0.56-1.14 | 0.220 |
| CC | 42 (17.6) | 70 (14.6) | 1.13 | 0.71-1.80 | 0.596 | 0.99 | 0.61-1.61 | 0.971 |
| rs3742106 |  |  |  |  |  |  |  |  |
| AA | 86 (35.4) | 166 (34.6) | 1.00 | reference | - | 1.00 | Reference | - |
| AC | 117 (48.1) | 234 (48.8) | 0.97 | 0.68-1.36 | 0.839 | 1.00 | 0.70-1.43 | 0.992 |
| CC | 40 (16.5) | 80 (16.7) | 0.96 | 0.61-1.53 | 0.880 | 1.06 | 0.65-1.71 | 0.823 |

aOdds ratio (OR) adjusted for age (categorical variable, using the global median age of 60 years as cutoff)
